# MD Simulations Revealing Special Activation Mechanism of Cannabinoid Receptor 1

**DOI:** 10.3389/fmolb.2022.860035

**Published:** 2022-03-29

**Authors:** Yiran Wu, Xuanxuan Li, Tian Hua, Zhi-Jie Liu, Haiguang Liu, Suwen Zhao

**Affiliations:** ^1^ iHuman Institute, ShanghaiTech University, Shanghai, China; ^2^ Complex Systems Division, Beijing Computational Science Research Center, Beijing, China; ^3^ School of Life Science and Technology, ShanghaiTech University, Shanghai, China

**Keywords:** cannabinoid receptor, CB1, molecular dynamics simulations, G protein-coupled receptor, activation mechanism, MD

## Abstract

Cannabinoid receptor 1 (CB1) is a G protein-coupled receptor (GPCR) that is gaining much interest for its regulating role in the central nervous system and its value as a drug target. Structures of CB1 in inactive and active states have revealed conformational change details that are not common in other GPCRs. Here, we performed molecular dynamics simulations of CB1 in different ligand binding states and with mutations to reveal its activation mechanism. The conformational change of the “twin toggle switch” residues F200^3.36^ and W356^6.48^ that correlates with ligand efficacy is identified as a key barrier step in CB1 activation. Similar conformational change of residues 3.36/6.48 is also observed in melanocortin receptor 4, showing this “twin toggle switch” residue pair is crucial for the activation of multiple GPCR members.

## Introduction

**FIGURE 1:**
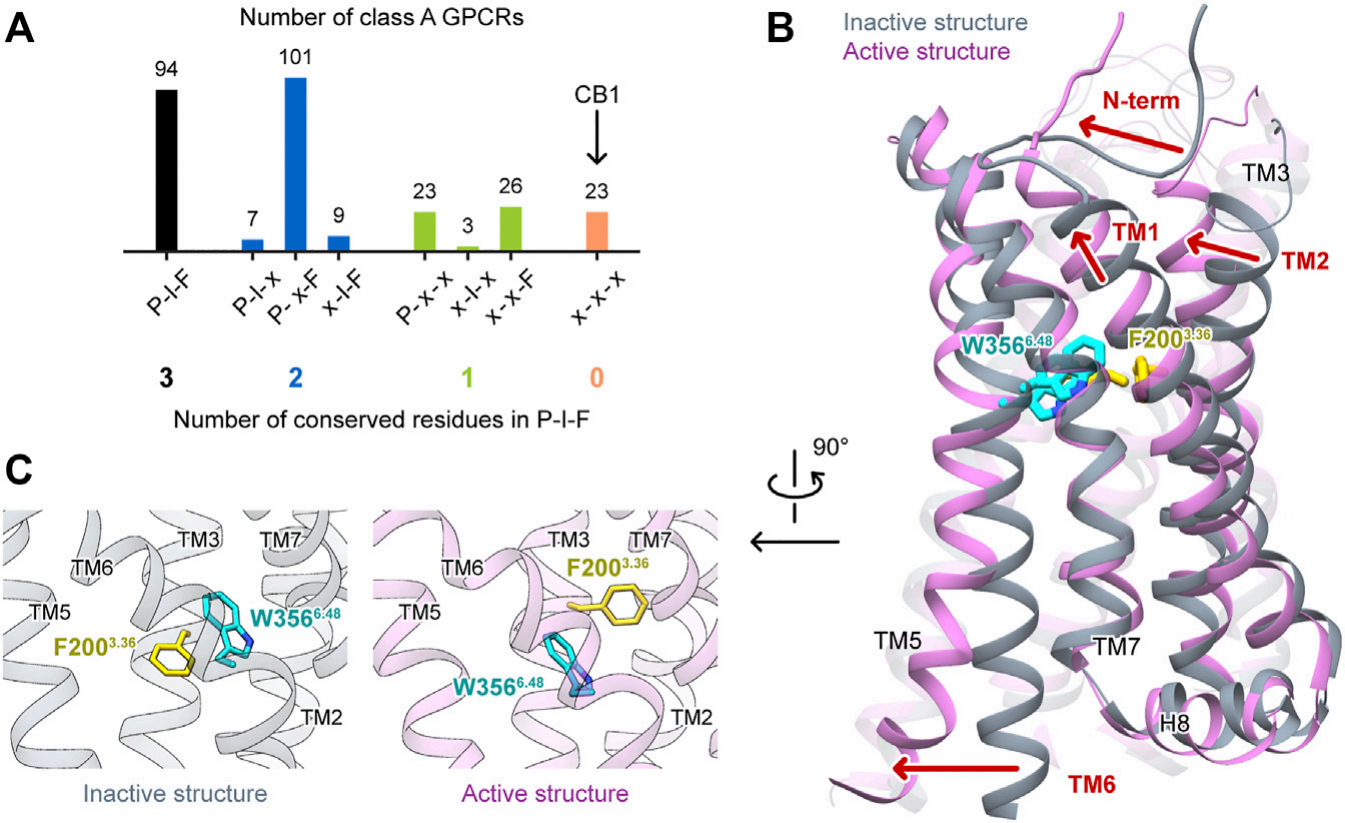
Special features of CB1 activations among class A GPCRs. **(A)** Conservativeness of the P-I-F motif in nonolfactory class A GPCRs (colored according to the number of conserved residues). CB1 is among the 23 members possessing none of P^5.50^, I^3.40^, and F^6.44^. **(B)** Comparison of CB1 inactive and active structures, showing movements of structural segments. **(C)** Side-chains of F200^3.36^ and W356^6.48^ in inactive and active structures of CB1 showed “twin toggle switch” of the two residues, a remarkable conformational change.

G protein-coupled receptors (GPCRs) constitute one of the largest protein families in the human genome and represent the largest class of drug targets (Hauser et al., 2017; Nieto Gutierrez and McDonald, 2018; Sriram and Insel, 2018). Typically, a GPCR detects molecules outside the cell with its extracellular part, and undergoes global conformational changes to transduce cell signaling. The molecular mechanism of GPCR activation has been intensively studied. In class A receptors, though the ligand binding sites are highly diverse, the movements of the cytoplasmic part during activation are similar, featuring the outward movement of TM6 and the distortion of TM7 to open the space for G protein binding (Rasmussen et al., 2011b; Latorraca et al., 2017). For the connector region, which links the extracellular part and the cytoplasmic part, the P^5.50^-I^3.40^-F^6.44^ [Ballesteros–Weinstein numbering (Ballesteros and Weinstein, 1995)] motif is a well-known microswitch (Rasmussen et al., 2011a; Wacker et al., 2013) that involves residues on three transmembrane (TM) helices. However, this P-I-F motif is not universal—among the 286 human nonolfactory class A GPCRs recorded in GPCRdb (Kooistra et al., 2021), only 94 possess this motif (Figure 1A). Therefore, GPCRs may have other mechanisms to relay the movements of the extracellular part to the cytoplasmic part.

Cannabinoid receptor 1 (CB1) is a GPCR that is highly expressed in the central nervous systems, and its endogenous ligands are a class of lipid mediators called endocannabinoids. It is also the major target of plant cannabis and synthetic cannabinoid drugs. CB1 has been extensively studied for ligand discovery, and its activation mechanism has also generated much attention. Interestingly, none of the three residues of the P-I-F motif is conserved in CB1. Instead, the residues in the three positions are L286^5.50^, V204^3.40^, and L352^6.44^. The structures of CB1 in the inactive (Hua et al., 2016; Shao et al., 2016) and active states (Hua et al., 2017; Krishna Kumar et al., 2019; Hua et al., 2020) exhibited movements of the extracellular part that were not observed in the other class A GPCRs (Figure 1B); TM1 and TM2 move inward during activation, shrinking the space of the orthosteric site (binding site of endogenous ligands), and the N-terminus forms a V-shaped loop and inserts into the orthosteric site in the inactive state, but lies straight outside the helix bundle in the active state. Furthermore, a remarkable conformational change of F200^3.36^ and W356^6.48^ was observed (Figure 1C). F200^3.36^ adopts different rotamers—in the inactive state, it points toward TM5, and in the active state, it points toward TM1. Upon activation, W356^6.48^ takes the space left by F200^3.36^, and correspondingly, the relative positions of TM3 and TM6 slide by 6.8 Å (measured by movement of W356^6.48^ Cα atom when TM3 is aligned). While this previously described conformational change of the “twin toggle switch” residues F200^3.36^/W356^6.48^ has been proposed to be the key for CB1 activation (Hua et al., 2017), its mechanism of action has not been investigated.

Molecular dynamics (MD) simulations have been widely used in the study of activation mechanisms of GPCRs (Dror et al., 2011; Miao et al., 2013; Kohlhoff et al., 2014; Yuan et al., 2014; Lee et al., 2015; Weis and Kobilka, 2018; Lovera et al., 2019; Wifling et al., 2019; Mattedi et al., 2020). For example, simulations of β2 adrenergic receptor (β2AR) produced the deactivation process and confirmed the key role of the P-I-F motif as a connector (Dror et al., 2011). Here, we performed microsecond MD simulations of CB1 in different conditions (Table 1) to investigate its special activation mechanism. We found that wild-type CB1, unlike β2AR, could not deactivate in MD simulations (three independent runs each for 1 µs). But when we introduced double mutation F200A/W356A to CB1, MD simulations captured the deactivation process, validating the conformational change of the “twin toggle switch” residues F200^3.36^/W356^6.48^ as a barrier step in the activation mechanism. Simulations with different types of ligands (four full agonists, a partial agonist, a neutral antagonist, and two inverse agonists) showed that ligand efficacy is correlated with the movement of F200^3.36^ and W356^6.48^. In addition, we analyzed the structures of other class A GPCRs and discovered the 3.36/6.48 “twin toggle switch” is not unique to CB1.

**TABLE 1:** Systems in MD simulations.

System number	Starting conformation	CB1 type	Ligand	Ligand type	Number of runs
#1	Active	Wild-type	None (apo)	—	3
#2	Active	F200A/W356A	None (apo)	—	4
#3	Active	Wild-type	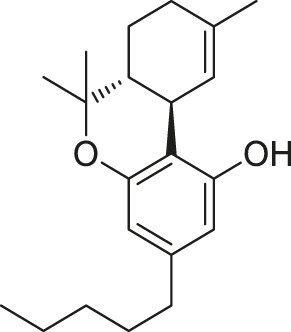 THC	Partial agonist	5
#4	Active	Wild-type	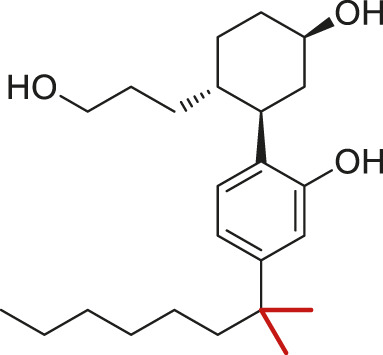 CP55,490^a^	Full agonist	5
#5	Active	Wild-type	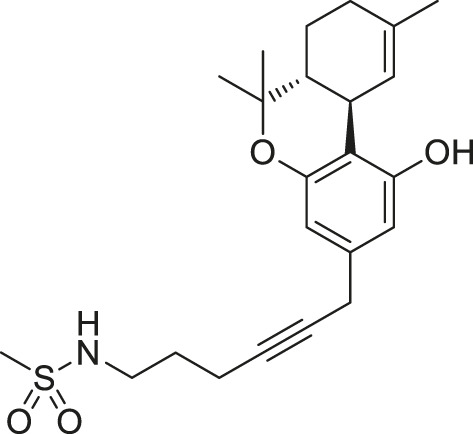 O-2050	Neutral antagonist	3
#6	Active	Wild-type	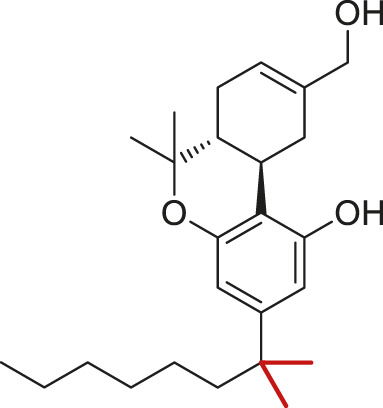 HU-210^a^	Full agonist	2
#7	Active	Wild-type	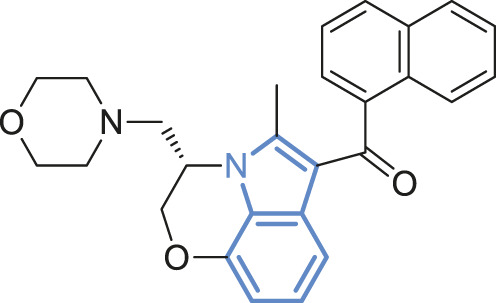 WIN55,212-2^b^	Full agonist	2
#8	Active	Wild-type	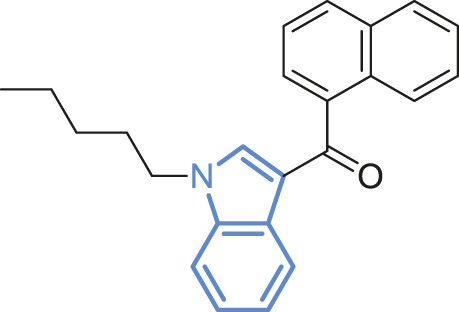 JWH018^b^	Full agonist	3
#9	Inactive	Wild-type	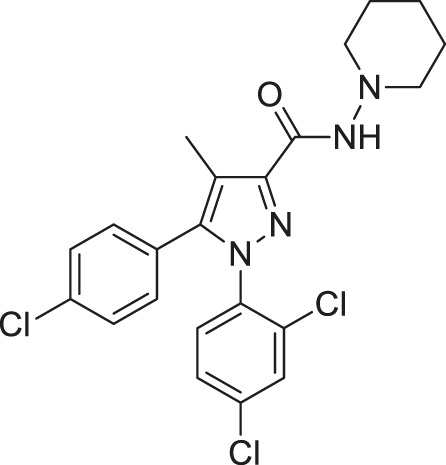 AM6538	Inverse agonist	2
#10	Inactive	Wild-type	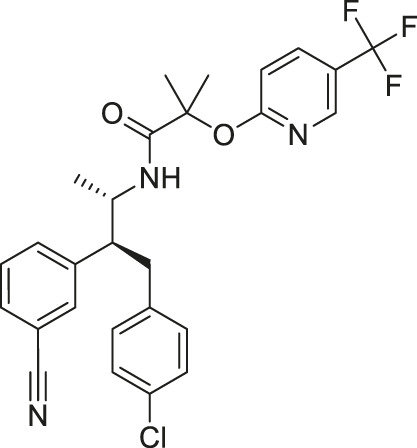 Taranabant	Inverse agonist	2

a C1′-gem-dimethyl group marked in red.

b Indole rings marked in blue.

## Materials and Methods

MD simulations were performed with GROMACS 5.1.2 (Abraham et al., 2015) using AMBER FF14SB force field (Maier et al., 2015). The crystal structures of CB1 were used as starting points—active state, PDB entry 5XRA (Hua et al., 2017) (apo or agonists/neutral antagonist-bound, systems #1–8 in Table 1), and the inactive state, PDB entries 5TGZ (Hua et al., 2016) (AM6538-bound, system #9 in Table 1) or 5U09 (Shao et al., 2016) (taranabant-bound, system #10 in Table 1). Protein processing was performed with the Protein Preparation Wizard tool in Maestro software of the Schrodinger platform v2015-4 (Schrödinger, 2015). In this step, the missing side-chain atoms were added, and the N- and C-termini were capped as acetyl and *N*-methylamide, respectively. Mutating back the four mutations in the CB1 crystal construct, adding N-terminal residues F102 and M103 for the active state model, and modeling the truncated third intracellular loop were done with the Residue and Loop Mutation tool. For CB1 double-mutant F200A/W356A (system 2 in Table 1), the alanine residues were generated manually by removing the side-chain atoms. Models of active/inactive CB1 were embedded into a pre-equilibrated lipid bilayer containing 180 POPC (1-palmytoil-2-oleoyl-sn-glycerol-3-phosphatidylcholine) molecules and 12,894 water molecules [originally generated by CHARMM-GUI (Jo et al., 2008)] using the membed tool in the GROMACS program. Sodium ions were added at 0.15 M in water, and chloride ions were added to neutralize the system. The constructed systems contained 154–155 POPC molecules and ∼12,300 water molecules, making the total number of atoms ∼63,000. Three-dimensional structures of ligands (except for crystallized AM6538 and taranabant) were generated using the LigPrep tool in Maestro, and their binding poses in CB1 were predicted by molecular docking performed with Glide v6.9 (Friesner et al., 2004; Halgren et al., 2004) in extra precision (Friesner et al., 2006). The topology files of all the ligands and the lipid POPC were generated using AmberTools in UCSF Chimera (Pettersen et al., 2004) version 1.10.2 and were converted to GROMACS format with the ACPYPE tool (Sousa da Silva and Vranken, 2012).

For each system, energy minimization was performed with position restraints on non–hydrogen atoms of the protein and ligand—first using the steepest descent algorithm until the maximum force was smaller than 1,000 kJ mol^−1^·nm^−1^, then using the conjugate gradient algorithm until the maximum force was smaller than 100 kJ mol^−1^·nm^−1^ or converged to machine precision. The initial velocity of each atom was generated at 310 K (2–5 times with different seeds), and the system was relaxed in NVT (constant Number of atoms, Volume, and Temperature) ensemble (310 K, Berendsen coupling scheme) for 300 ps (time step 1 fs). The system was then equilibrated with position restraints to non–hydrogen atoms of protein and ligand in NPT (constant Number of atoms, Pressure, and Temperature) ensemble (310 K, 1 atm semi-isotropic, both Berendsen coupling scheme) for 15 ns in total—first with force constant 1,000 kJ mol^−1^·nm^−2^ for 5 ns (time step 1 fs), then gradually reducing the restraint force following 800, 600, 400, 200, and 0 kJ mol^−1^·nm^−2^, each for 2 ns (time step 2 fs). Finally, productive simulations were performed in the NPT ensemble (310 K, Nose–Hoover coupling scheme; 1 atm semi-isotropic, Parrinello–Rahman coupling scheme) for 1 μs. For all the simulations (relaxing, balancing, and productive), the leap-frog algorithm was used as the integrator, and constraints were set on the bonds with hydrogen atoms using the LINCS method; the cut-off distance for Van der Waals and short-range electrostatic interactions were set to 10 Å; and long-range electrostatic interactions were computed using smooth particle mesh Ewald method.

Snapshots were taken every 1 ns for analysis. The reference structures were—active state, cryo-EM structure of CB1/AM841/G protein (PDB entry 6KPG [Hua et al., 2020)] and inactive state, crystal structure of CB1/AM6538 [PDB entry 5TGZ (Hua et al., 2016)]. The overall root mean square deviations (RMSD) were calculated based on the Cα atoms of residues 106–306 and 335–411 (the third intracellular loop was neglected).

## Results

### Fluctuation of F200^3.36^/W356^6.48^ Side-Chains

**FIGURE 2:**
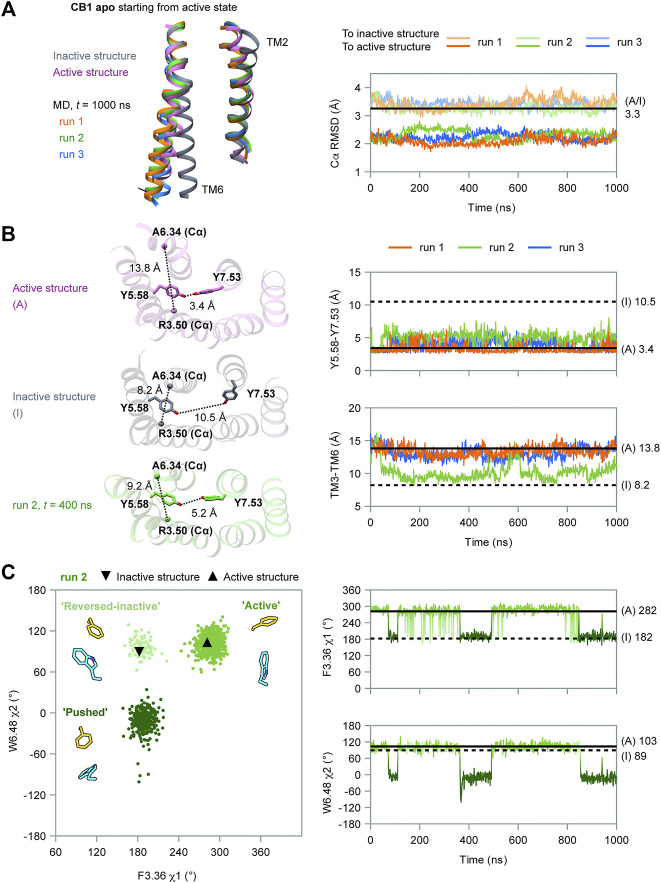
MD simulation results of apo CB1 starting from the active state. **(A)** Overall structure did not show the trend of deactivation during simulations. Left panel: the ending conformations of the three runs resemble the active structure (only TM2 and TM6 are shown for the sake of clarity). Right panel: root mean square deviations (RMSD) of Cα atoms showed CB1 remained more similar to the active structure than to the inactive through the three runs. (A/I) noted for RMSD of Cα atoms between the active structure and inactive structure. **(B)** Run 2 captured movements of the cytoplasmic part. Left panel: indices Y5.58-Y7.53 and TM3-TM6 in inactive structure (I), active structure (A), and representative snapshot in run 2. Right panel: Y5.58-Y7.53 distance remained around the value in (A); TM3-TM6 distance remained around (A) in runs 1 and 3, but varied between (A) and (I) in run 2. **(C)** Side-chain conformations of F200^3.36^ and W356^6.48^ in run 2. Left panel: distribution of dihedral angles showed three configurations of F200^3.36^/W356^6.48^, as illustrated by representative snapshots. In the configuration “reversed-inactive,” the dihedral angles were close to values in the inactive state, but the positions of the two residues were still the same as in the active state. Right panel: F200^3.36^ χ1 and W356^6.48^ χ2 during simulation (results for runs 1 and 3 are shown in Supplementary Figure S2).

First, we performed MD simulations of wild-type, apo CB1 without the ligand, starting from the active state (system #1 in Table 1). Three independent simulations were carried out, each for 1 μs. At the overall structure level, CB1 remained in the active state and did not show trends of deactivation (Figure 2A). According to the RMSD of the Cα atoms, all through the simulations, the conformations of CB1 were more similar to the active structure than to the inactive structure.

However, the movement of the cytoplasmic part showed one run among the three, run 2, deviated from the initial active state. We used two indicators to analyze the movement of this part (Figure 2B): 1) the distance between Y294^5.58^ and Y397^7.53^, the two conserved residues in class A GPCRs that lay far apart in the inactive state but form interactions in the active state (10.5 and 3.4 Å, respectively, for CB1, marked by side-chain Oη atoms), displaying the distortion of TM7; and 2) the distance between the cytoplasmic ends of TM3 and TM6, as loosening of the two segments is a key movement upon activation (8.2 Å in the inactive state and 13.8 Å in the active state for CB1, marked by R214^3.50^ and A342^6.34^ Cα atoms). Y294^5.58^ and Y397^7.53^ remained close in all three runs, revealing an active feature. The cytoplasmic ends of TM3 and TM6 remained far apart in runs 1 and 3, revealing an active feature in the two runs. But in run 2, the cytoplasmic ends showed larger changes: the TM3-TM6 distance first decreased from ∼14 to ∼9 Å, close to the value of the inactive state at 43 ns. It kept this distance until ∼500 ns and varied between ∼9 and ∼14 Å, corresponding to the inactive and active states during the remaining 500 ns. For β2AR, the MD simulations suggested that distortion of TM7 occurred at the early stage of the deactivation process and indicated a transition to the intermediate state, while TM3-TM6 became close later and indicated a transition to the inactive state (Dror et al., 2011) (schematic diagram in Supplementary Figure S1A). Comparing to β2AR, our simulations of CB1 did not show transitions to either inactive state or intermediate state (Supplementary Figure S1B,C). Therefore, the changes of TM3-TM6 distance captured in run 2 were the local movements of the cytoplasmic part.

Interestingly, only run 2, the run with the local movement of the cytoplasmic part, revealed remarkable conformational changes of the “twin toggle switch” residues (Figure 2C and Supplementary Figure S2). We computed F200^3.36^ χ1 and W356^6.48^ χ2, two side-chain dihedral angles that can display the conformational changes. In runs 1 and 3, F200^3.36^ χ1 and W356^6.48^ χ2 generally remained close to their initial values, with changes for only short times (Supplementary Figure S2). In run 2, the changes of the two dihedral angles could last long (Figure 2C)—F200^3.36^ could adopt the rotamer of the active or inactive states; W356^6.48^, which has the same rotamer in the active and inactive structures, could adopt a new conformation during the simulation. Three configurations can be defined according to the distribution of the two dihedral angles: “active” (close to the conformation in the active structure), “reversed-inactive” (where dihedral angles were close to the values in the inactive state, but the positions of the two residues were still reversed), and “pushed”(where W356^6.48^ was pushed to the new rotamer by F200^3.36^). “Active” was the most frequently occurring configuration; the receptor could also stay in “pushed” configuration for more than 100 ns, but for only a very short time in “reversed-inactive.” Though F200^3.36^ and W356^6.48^ fluctuated among the three configurations, the bulky side-chains could not switch their locations. This behavior suggests that the conformational change of the “twin toggle switch” residues F200^3.36^/W356^6.48^ might be a key barrier step in the deactivation process.

### Capturing CB1's Deactivation Process

**FIGURE 3:**
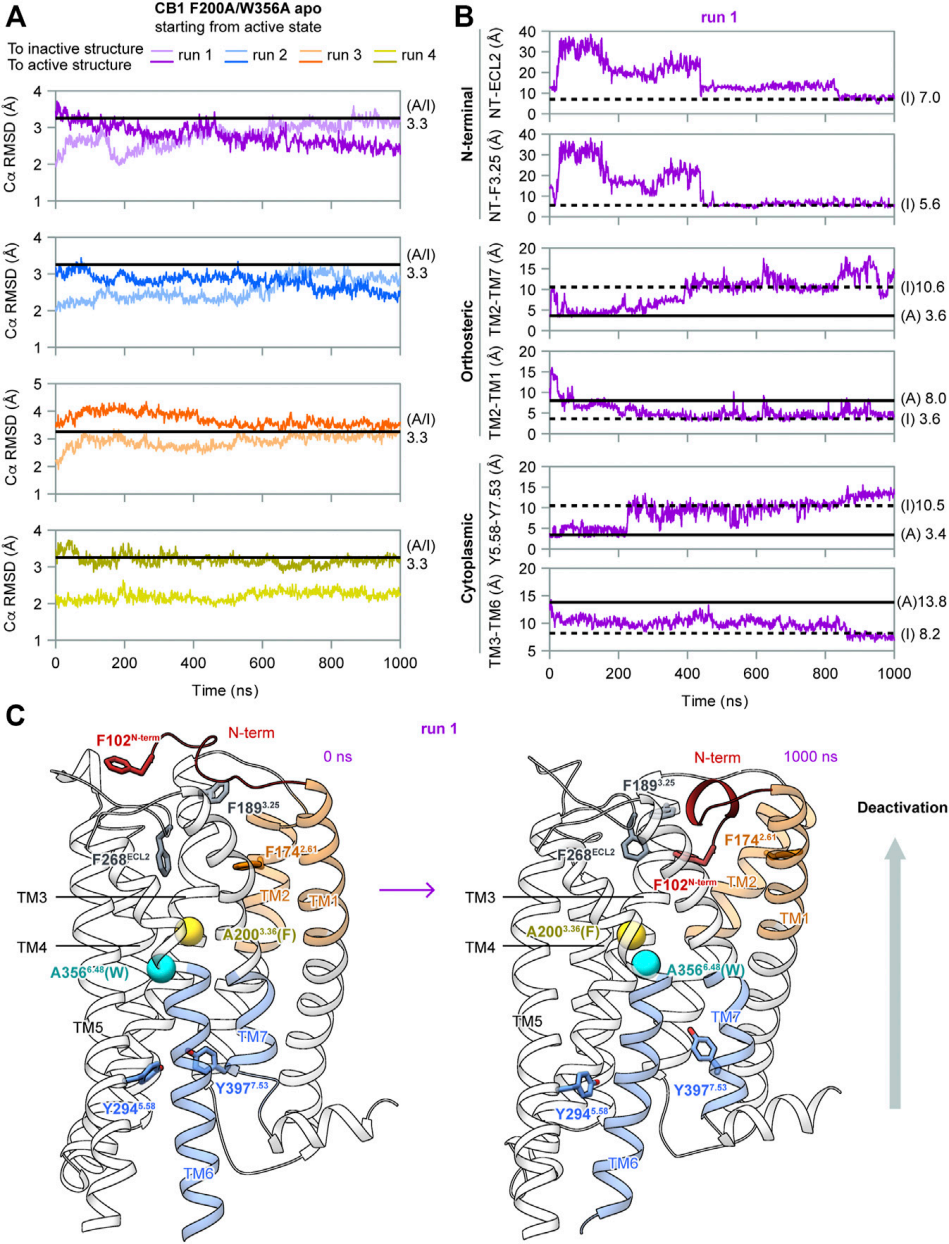
MD simulation results of CB1 double-mutant F200A/W356A starting from the active state. **(A)** RMSD of Cα atoms to inactive or active structures showed the overall structure gradually shifted from the active state to inactive state in runs 1–2. **(B)** Indicators for movements at different parts of CB1 in run 1, showing deactivation transitions started at the cytoplasmic end and finalized at the extracellular end. The two N-terminal indicators could not be measured in the active structure because F102^N−term^ was not solved. Results for run 2 are shown in Supplementary Figure S3. **(C)** Structures of the first and the last snapshot of run 1, showing conformational changes at different parts of CB1 during simulation. Cβ atoms of A200^3.36^ (phenylalanine in wild type CB1) and A356^6.48^ (tryptophan in wild type CB1) are shown as spheres.

To determine the role of F200^3.36^ and W356^6.48^ in the activation of CB1, we performed MD simulations of apo CB1 with double mutation F200A/W356A, starting from the same active state (system #2 in Table 1). Among the four independent 1 μs runs carried out, two (runs 1–2) captured deactivation transitions (Figure 3). During simulations of runs 1–2, the RMSD of Cα atoms to the inactive structure decreased to less than that to the active structure, which increased during simulations, thus showing that the overall structure gradually shifted from the active state to the inactive state (Figure 3A). These results supported that the conformational change of the “twin toggle switch” F200^3.36^/W356^6.48^ was a barrier step, for we observed the deactivation process when these bulky side-chains were removed.

In our simulations, deactivation of CB1 initiated from the cytoplasmic end to the extracellular end (Figures 3B, 3C, and Supplementary Figure S3). To analyze the movements at different parts of CB1, we introduced four more indicators: Two at the orthosteric pocket to display the inward movement of TM1/2 (TM2-TM1, marked by the distance between the F174^2.61^ side-chain Cζ atom and A120^1.36^ main-chain O atom; TM2-TM7, marked by the distance between the F174^2.61^ side-chain Cζ atom and A380^7.36^ main-chain O atom). Two at the N-terminal region to display insertion of the N-terminus into the orthosteric pocket (NT-F3.25, marked by the distance between the centers of the benzene rings of F102^N−term^ and F189^3.25^; NT-ECL2, marked by the distance between the centers of the side-chain benzene rings of F102^N−term^ and F268^ECL2^). The values of the two N-terminal indicators in the active structure could not be obtained because F102^N−term^ was not solved. However, it is certain that in the active state, F102^N−term^ is far away from F189^3.25^ and F268^ECL2^ because the N-terminus lies outside the helix bundle in the active state and the two residues are inside the orthosteric pocket. In run 1 (Figure 3B), TM3-TM6 became close soon after the simulation started. At ∼200 ns, distortion of TM7 occurred (as Y5.58-Y7.53 moving apart) and TM2-TM1 moved close. Transitions of the other three indicators occurred at ∼400 ns, and around this time, the overall structure became more similar to the inactive structure than to the active structure (Figure 3A). Finally at ∼800 ns, F102^N-term^ became more close to F268^ECL2^, showing the N-terminus inserted deeply into the pocket. At the same time, indicators of the orthosteric pocket and the cytoplasmic part fluctuated, showing movements of different regions of the receptor are correlated. Especially, TM3-TM6 distance shifted to the value in the inactive state at this stage, suggesting the N-terminus inserting into the pocket is correlated to the transition to the fully inactive state (Figure 3B). By the end of the simulation, conformation of CB1 highly resembled the inactive structure (Figure 3C). In run 2 (Supplementary Figure S3), it was similar that transitions at the cytoplasmic part were earlier than the transitions at the orthosteric pocket and N-terminal region, though the final insertion of N-terminus into the orthosteric pocket was not captured, as revealed by indicator NT-ECL2.

### Ligand Efficacy Correlates With the Movement of F200^3.36^/W356^6.48^ Side-Chains

**FIGURE 4:**
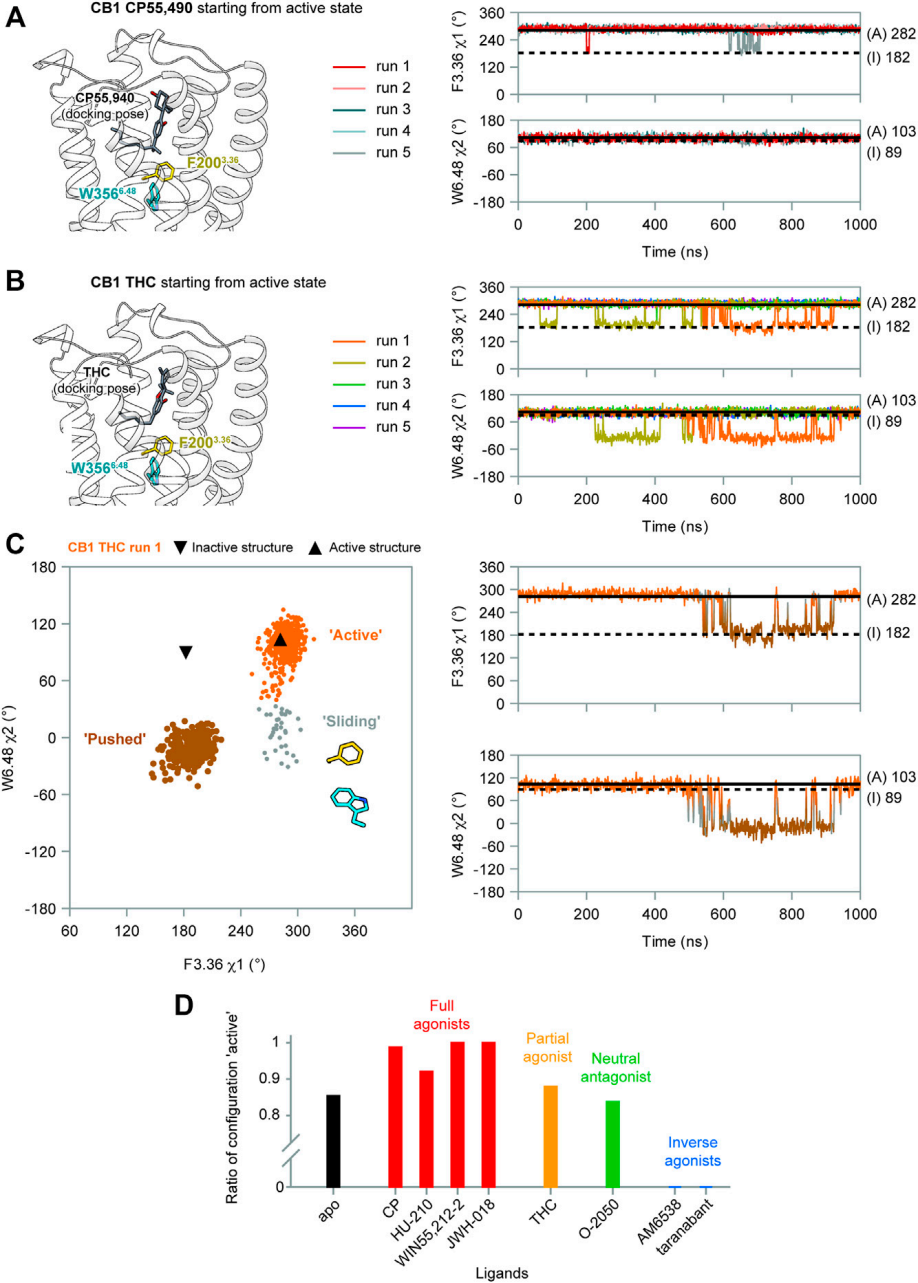
F200^3.36^/W356^6.48^ side-chain conformations during MD simulations of CB1 bound with CP55,940 and THC. **(A)** In CP55,940-bound CB1, F200^3.36^ and W356^6.48^ side-chains generally remained stable during simulations. **(B)** THC-bound CB1 showed fluctuations of F200^3.36^ and W356^6.48^ side-chains in runs 1–2. **(C)** Movements of F200^3.36^ and W356^6.48^ in THC-bound CB1 run 1 (results for run 2 are shown in Supplementary Figure S4A). Left panel: distribution of dihedral angles showed a new configuration of F200^3.36^/W356^6.48^. Right panel: F200^3.36^ χ1 and W356^6.48^ χ2 during simulation. **(D)** The ratio of configuration “active” in all the simulations of CB1 bound to each ligand, showing the correlation with ligand efficacy.

(−)-*trans*-Δ^9^-tetrahydrocannabinol (THC) is the principal active component of plant cannabis and acts as a partial agonist of CB1 *in vitro*; CP55,940, a synthetic analog of THC, acts as a full agonist (Paronis et al., 2012). The C1′-gem-dimethyl substitution in CP55,940 (Table 1) is key for the enhanced efficacy (Makriyannis and Rapaka, 1987), and this moiety forms hydrophobic interactions with F200^3.36^ in the active state (Figure 4A). To study how the “twin toggle switch” residues F200^3.36^/W356^6.48^ were affected by ligands with different efficacies, we performed MD simulations of CB1 bound with THC and CP55,940 (systems #3–4 in Table 1). For each system, five independent 1-μs simulations were performed.

Our results show that the full agonist CP55,490 stabilized the “twin toggle switch” residues in the active state (Figure 4A)—F200^3.36^ χ1 only shifted temporarily in two runs, while W356^6.48^ χ2 did not change at all, suggesting no fluctuation was observed in all the five runs. By contrast, for the partial agonist THC-bound CB1, two runs (1–2) captured fluctuations of the “twin toggle switch” residues (Figure 4B). The distribution of the dihedral angles of F200^3.36^ χ1 and W356^6.48^ χ2 exhibited a configuration, which we named “sliding,” that did not appear in the simulations of apo CB1 (Figure 4C and Supplementary Figure S4A). Here, the relative positions of the side-chains did not switch, but the main-chain of W356^6.48^ slid toward the position in the inactive state, with F200^3.36^ χ1 remaining but W356^6.48^ χ2 shifting (Figure 4C).

To further test the relationship between ligand efficacy and the movement of F200^3.36^ and W356^6.48^, we included several more ligands with different efficacies on CB1 for our MD simulations (systems #5–10 in Table 1): neutral antagonist O-2050 (three runs); full agonists HU-210, WIN55,212-2, and JWH-018 (each for two to three runs); and inverse agonists AM6538 and taranabant (each for two runs). For the neutral antagonist O-2050-bound CB1, one of the three runs captured the fluctuations of F200^3.36^ and W356^6.48^ side-chains (Supplementary Figure S5). Interestingly, the configuration “sliding” occurred very frequently in this run while “reversed-inactive” occurred only for a few snapshots. The full agonists, similar to CP55,940, stabilized CB1 in the active state (Supplementary Figure S6), excepting one run of HU-210–bound CB1 had a shifted F200^3.36^ χ1 for ∼200 ns. Both inverse agonists, by contrast, stabilized CB1 in the inactive state (Supplementary Figure S7). We counted the ratio of the “active” configuration through all the simulations of each ligand (or apo)—the values are close to 1 for full agonists, ∼0.85 for partial agonist, neutral antagonist, or apo, and 0 for inverse agonists (Figure 4D). These results confirmed that ligand efficacy is determined by the movement of the “twin toggle switch” residues, F200^3.36^ and W356^6.48^, upon being bound with the ligand.

### The 3.36/6.48 “Twin Toggle Switch” Is Not Unique to CB1

**FIGURE 5:**
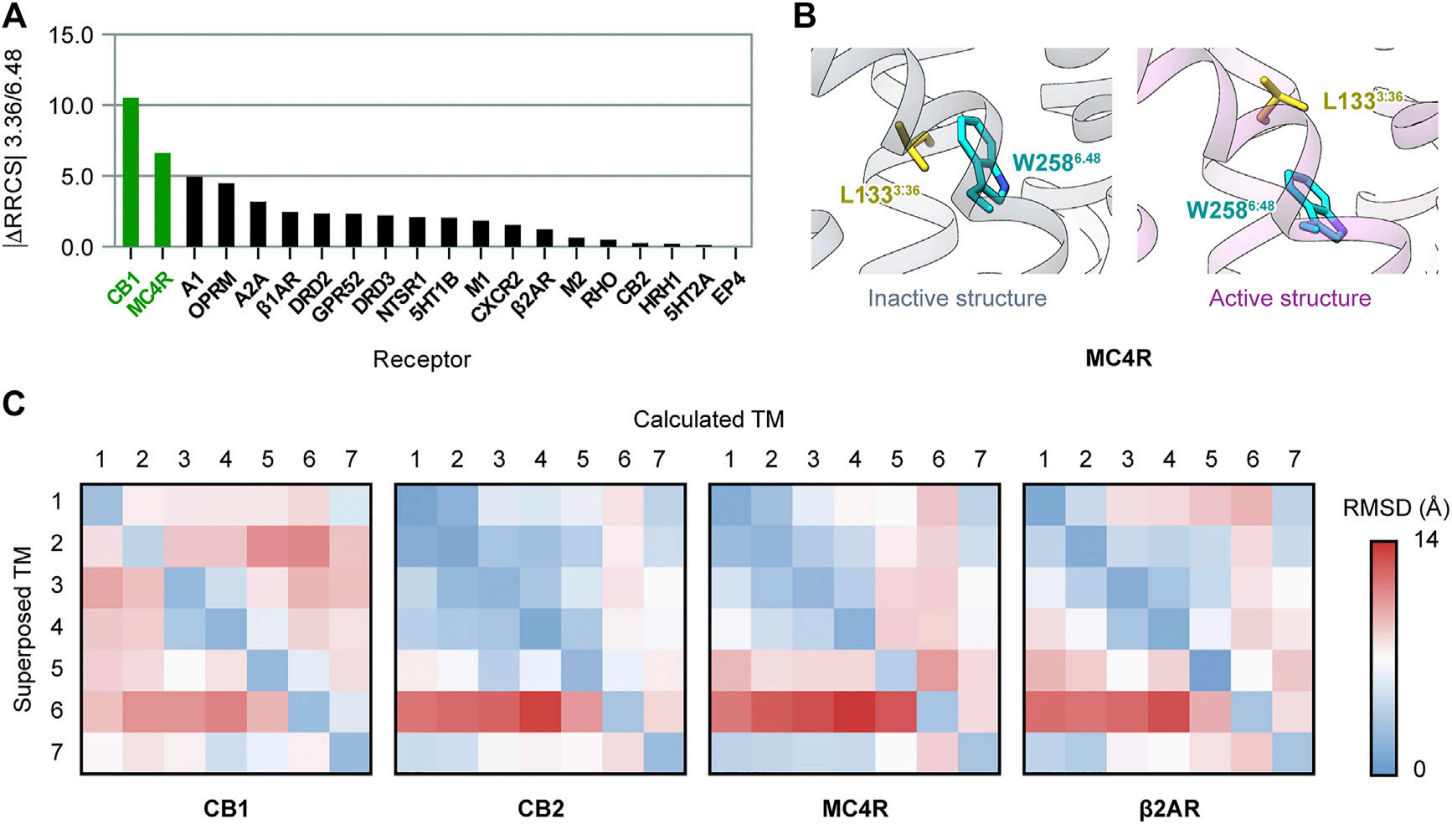
Comparison of activation mechanisms among class A GPCRs. **(A)** Difference of 3.36/6.48 residue–residue contact score (RRCS) in inactive and active structures in class A GPCRs. The top two ranked receptors, CB1 and MC4R, have the “twin toggle switch” of 3.36/6.48. **(B)** “Twin toggle switch” of L133^3.36^ and W258^6.48^ MC4R. **(C)** 7 × 7 RMSD matrices of inactive/active structure pairs for class A GPCRs. Results for CB1, its closest homolog—CB2, the only other receptor containing “twin toggle switch”—MC4R, and prototype GPCR—β2AR are shown here; results for the 16 other GPCRs are shown in Supplementary Figure S9.

We also investigated whether there are other class A GPCRs with the “twin toggle switch” of 3.36/6.48 activation mechanism. Until June 2021, there were 20 class A GPCRs with both inactive and active structures solved (Supplementary Table S1). For each receptor, we evaluated the change of 3.36/6.48 interactions upon activation using the residue–residue contact score (RRCS), a method based on atomic distance to quantify the strength of contact between residue pairs (Zhou et al., 2019). The value |ΔRRCS| (difference of RRCS in inactive and active structures, absolute value) will be larger if the 3.36/6.48 interactions are more different in inactive and active structures. The evaluating results (Figure 5A) revealed CB1 is the top-ranked GPCR for different 3.36/6.48 interactions in the inactive and active states. The second ranked receptor, melanocortin receptor 4 (MC4R), also possesses the “twin toggle switch,” though the residue at position 3.36 is not phenylalanine but leucine (Figure 5B). The conformational change of the “twin toggle switch” residues at 3.36/6.48 does not occur in other GPCRs, including the two with the same F3.36/W6.48 as in CB1—cannabinoid receptor 2, CB2 (Li et al., 2019; Hua et al., 2020), and C-X-C chemokine receptor type 2, CXCR2 (Liu et al., 2020) (Supplementary Figure S8).

We further examined conformational changes of the overall structure upon activation in class A GPCRs, but found no correlation with “twin toggle switch” 3.36/6.48. The 7 × 7 RMSD matrix is a method to compare two GPCR structures by aligning one TM helix and computing RMSD of the seven TM helices (Wang et al., 2017). We constructed 7 × 7 RMSD matrices between inactive/active structure pairs of the 20 GPCRs (Figure 5C and Supplementary Figure S9). The results showed CB1 has a special inter-helical movement pattern, with relative movements between most helix pairs (Figure 5C), while movements are confined to very few helices in all the other GPCRs including MC4R (Figure 5C and Supplementary Figure S9). These results exhibited that the conformational changes of CB1 during activation are distinct among class A GPCRs, though “twin toggle switch” 3.36/6.48 is not unique to CB1.

## Discussion

**FIGURE 6:**
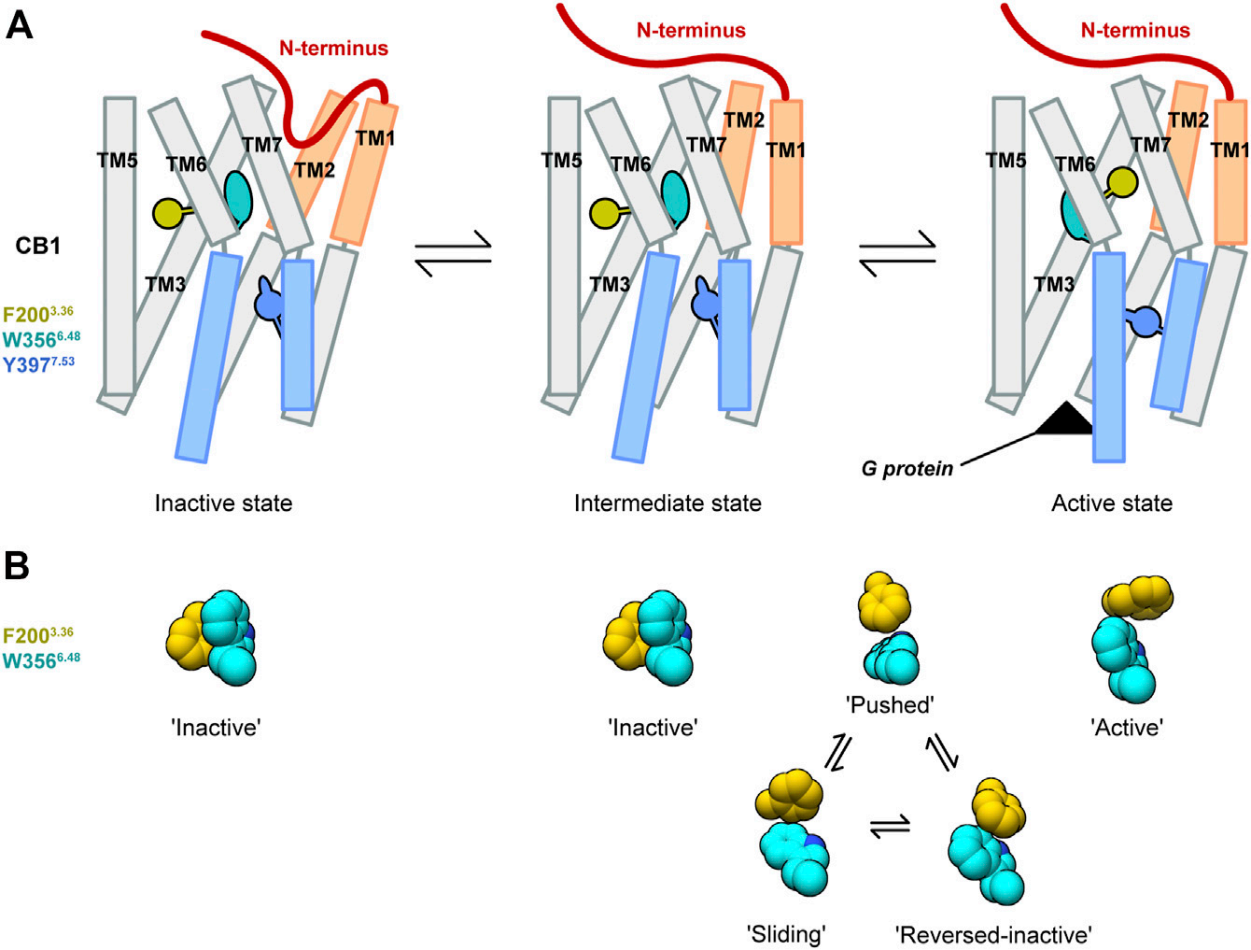
Model of the CB1 activation mechanism. **(A)** Showed the overall CB1 receptor. **(B)** Showed configurations of the “twin toggle switch” residues F200^3.36^ and W356^6.48^.

Structures of CB1 in inactive and active states showed uncommon conformational changes. Our MD simulations of deactivation process of CB1 captured conformational transitions at different regions. Based on these results, we propose a model of the special activation mechanism of CB1 (Figure 6).

There is an intermediate state of CB1 that is similar to the active structure at the extracellular part, but similar to the inactive structure at the cytoplasmic part. This is supported by our MD simulations starting from apo CB1 in the active state: for wild-type CB1, we observed movements of only the cytoplasmic part; for CB1 double-mutant F200A/W356A, transitions to the inactive state occurred earlier at the cytoplasmic part than the extracellular part. In the intermediate state, cytoplasmic ends of TM3 and TM6 were close, occupying the space for G protein binding. Eventually, such conformation of CB1 was already obtained in the experiment recently—in the crystal structure of CB1 bound with CP55,940 and ORG27569 (an allosteric modulator that enhances the binding of CP55,940 but reduces its signaling (Price et al., 2005; Shao et al., 2019).

This activation mechanism of CB1 is uncommon. For most class A GPCRs, the extracellular parts adopt similar conformations when bound with agonists or antagonists (Hua et al., 2017). Therefore, receptors with large-scale movement around the orthosteric pocket, including CB1 and P2Y purinoceptor 12 (Jin Zhang et al., 2014; Kaihua Zhang et al., 2014), may have special mechanisms for the coupling of the orthosteric pocket and the G protein–binding site. Moreover, a continuous water channel is an important hallmark for GPCR activation discovered by MD simulations (Yuan et al., 2013; Yuan et al., 2014; Yuan et al., 2015). However, we did not observe such a water channel in our simulations. This may be because the orthosteric pocket of CB1 is highly hydrophobic thus few water molecules could enter. We speculate this type of “dry activation” occurs in all the lipid receptors with highly hydrophobic orthosteric pockets similar to CB1.

The conformational change of the “twin toggle switch” residues F200^3.36^/W356^6.48^ occurs during transition from the intermediate state to active state. In our MD simulations of wild-type CB1, though the intermediate state was not achieved due to limited simulation time, we observed fluctuations of F200^3.36^/W356^6.48^ side-chains accompanied with movements of the cytoplasmic part, corresponding to the difference between the intermediate state and active state. Furthermore, in the structure of CB1 stabilized in the intermediate state by CP55,940 and ORG27569-bound (Shao et al., 2019), F200^3.36^/W356^6.48^ adopts the “inactive” conformation. For the process of this conformational change, we identified three possible transient configurations of F200^3.36^/W356^6.48^: “reversed-inactive,” “pushed,” and “sliding.” Among these configurations, “pushed” occurred most frequently and were observed in all the runs that captured fluctuations of F200^3.36^/W356^6.48^, thus might be a major configuration during the transition between the intermediate state and active state. For the other two configurations, run 2 of the apo and run 2 of the THC-bound CB1 mainly sampled “reversed-inactive” (Figure 2C and Supplementary Figure S4A), while run 1 of the THC-bound and run 3 of the O-2050-bound CB1 sampled more “sliding” than “reversed-inactive” (Figure 4C, Supplementary Figure S5B). So, we cannot reach any solid conclusions to discriminate partial agonist against neutral antagonist based on the limited MD simulations. The whole process of conformational changes of the “twin toggle switch” residues is beyond the time scale of this study and might be obtained with accelerated MD simulations or super long time conventional MD simulations. Also, free energy calculation in future investigations would help to obtain the energy barrier of the conformational change of “twin toggle switch” residues and decide the path of activation.

The role of conserved W6.48 in class A GPCR activation has long been debated. W6.48 is called the “toggle switch” because it was proposed to adopt different side-chain rotamers in the inactive and active states of β2AR by Monte Carlo simulations (Shi et al., 2002). But the crystal structure of the active state β2AR showed no change of W6.48 rotamer (Rasmussen et al., 2011a). In most structures of class A GPCRs, either inactive or active, W6.48 adopts the same rotamer as in β2AR, contradicting W6.48 as the “toggle switch” of common activation mechanism. However, there are several receptors showing W6.48 with different side-chain rotamers: 5-hydroxytryptamine receptor 2A (Kim et al., 2020) and μ-opioid receptor (Koehl et al., 2018) have unusual W6.48 in the active state; muscarinic acetylcholine receptors M1 (Thal et al., 2016), M2 (Suno et al., 2018), and CB2 (Li et al., 2019) have unusual W6.48 in the inactive state. These suggested there are diverse activation mechanisms of class A GPCRs at the connector region. “Twin toggle switch” of residues 3.36/6.48 in CB1 and MC4R provides a special mechanism and expands our understanding of the activation of GPCRs.

## Data Availability

The original contributions presented in the study are included in the article/Supplementary Material, further inquiries can be directed to the corresponding authors.
