# Supplementary material for: MD Simulations Revealing Special Activation Mechanism of Cannabinoid Receptor 1
Source: Front Mol Biosci. 2022 Mar 29;9:860035. doi: 10.3389/fmolb.2022.860035 (PMC9004671; doi:10.3389/fmolb.2022.860035)
Supplement: Supplementary file 1 [file DataSheet1.PDF]

Supplementary Material

Table S1. List of 20 class A receptors with inactive and active structures solved.

Name	Abbreviation	Species	PDB Entries	
			Inactive	Active
Cannabinoid receptor 1	CB1	Human	5TGZ (Hua et al., 2016)	6KPG (Hua et al., 2020)
Cannabinoid receptor 2	CB2	Human	5ZTY (Li et al., 2019)	6KPF (Latorraca et al., 2017)
β2 adrenergic receptor	β2AR	Human	2RH1 (Cherezov et al., 2007)	3SN6 (Rasmussen et al., 2011)
β1 adrenergic receptor	β1AR	Turkey	2VT4 (Warne et al., 2008)	7JJO (Su et al., 2020)
Serotonin receptor 1B	5HT1B	Human	5V54 (Yin et al., 2018)	6G79 (Garcia-Nafria et al., 2018b)
Serotonin receptor 2A	5HT2A	Human	6A94 (Kimura et al., 2019)	6WHA (Kim et al., 2020)
Dopamine receptor D2	DRD2	Human	6CM4 (Wang et al., 2018)	7JVR (Zhuang et al., 2021)
Dopamine receptor D3	DRD3	Human	3PBL (Chien et al., 2010)	7CMV (Xu et al., 2021)
Histamine receptor H1	HRH1	Human	3RZE (Shimamura et al., 2011)	7DFL (Xia et al., 2021)
Muscarinic acetylcholine receptor M1	M1	Human	5CXV (Thal et al., 2016)	6OIJ (Maeda et al., 2019)
Muscarinic acetylcholine receptor M2	M2	Human	5ZK8 (Suno et al., 2018)	6OIK (Maeda et al., 2019)
Adenosine receptor A1	A1	Human	5UEN (Glukhova et al., 2017)	6D9H (Draper-Joyce et al., 2018)
Adenosine A2A receptor	A2A	Human	4EIY (Liu et al., 2012)	6GDG (Garcia-Nafria et al., 2018a)
Prostaglandin E2 receptor EP4	EP4	Human	5YWY (Toyoda et al., 2019)	7D7M (Nojima et al., 2021)
Rhodopsin	RHO	Bovine	1U19 (Okada et al., 2004)	6OYA (Gao et al., 2019)
Melanocortin receptor 4	MC4R	Human	6W25 (Yu et al., 2020)	7AUE (Israeli et al., 2021)
Neurotensin receptor type 1	NTSR1	Rat	6ZIN (Deluigi et al., 2021)	7L0P (Zhang et al., 2021)
Mu-type opioid receptor	OPRM	Mouse	4DKL (Manglik et al., 2012)	6DDF (Koehl et al., 2018)
C-X-C chemokine receptor type 2	CXCR2	Human	6LFL (Liu et al., 2020)	6LFM (Liu et al., 2020)
G protein-coupled receptor 52	GPR52	Human	6LI1 (Lin et al., 2020)	6LI3 (Lin et al., 2020)

References

Cherezov, V., Rosenbaum, D.M., Hanson, M.A., Rasmussen, S.G., Thian, F.S., Kobilka, T.S., et al. (2007). High-resolution crystal structure of an engineered human beta2-adrenergic G protein-coupled receptor. *Science* 318(5854)**,** 1258-1265. doi: 10.1126/science.1150577.

Chien, E.Y., Liu, W., Zhao, Q., Katritch, V., Han, G.W., Hanson, M.A., et al. (2010). Structure of the human dopamine D3 receptor in complex with a D2/D3 selective antagonist. *Science* 330(6007), 1091-1095. doi: 10.1126/science.1197410.

Deluigi, M., Klipp, A., Klenk, C., Merklinger, L., Eberle, S.A., Morstein, L., et al. (2021). Complexes of the neurotensin receptor 1 with small-molecule ligands reveal structural determinants of full, partial, and inverse agonism. *Sci Adv* 7(5). doi: 10.1126/sciadv.abe5504.

Draper-Joyce, C.J., Khoshouei, M., Thal, D.M., Liang, Y.L., Nguyen, A.T.N., Furness, S.G.B., et al. (2018). Structure of the adenosine-bound human adenosine A1 receptor-Gi complex. *Nature* 558(7711), 559-563. doi: 10.1038/s41586-018-0236-6.

Dror, R.O., Arlow, D.H., Maragakis, P., Mildorf, T.J., Pan, A.C., Xu, H., et al. (2011). Activation mechanism of the beta2-adrenergic receptor. *Proc Natl Acad Sci U S A* 108(46), 18684-18689. doi: 10.1073/pnas.1110499108.

Gao, Y., Hu, H., Ramachandran, S., Erickson, J.W., Cerione, R.A., and Skiniotis, G. (2019). Structures of the Rhodopsin-Transducin Complex: Insights into G-Protein Activation. *Mol Cell* 75(4), 781-790 e783. doi: 10.1016/j.molcel.2019.06.007.

Garcia-Nafria, J., Lee, Y., Bai, X., Carpenter, B., and Tate, C.G. (2018a). Cryo-EM structure of the adenosine A2A receptor coupled to an engineered heterotrimeric G protein. *Elife* 7. doi: 10.7554/eLife.35946.

Garcia-Nafria, J., Nehme, R., Edwards, P.C., and Tate, C.G. (2018b). Cryo-EM structure of the serotonin 5-HT1B receptor coupled to heterotrimeric Go. *Nature* 558(7711), 620-623. doi: 10.1038/s41586-018-0241-9.

Glukhova, A., Thal, D.M., Nguyen, A.T., Vecchio, E.A., Jorg, M., Scammells, P.J., et al. (2017). Structure of the Adenosine A1 Receptor Reveals the Basis for Subtype Selectivity. *Cell* 168(5), 867-877 e813. doi: 10.1016/j.cell.2017.01.042.

Hua, T., Li, X., Wu, L., Iliopoulos-Tsoutsouvas, C., Wang, Y., Wu, M., et al. (2020). Activation and Signaling Mechanism Revealed by Cannabinoid Receptor-Gi Complex Structures. *Cell* 180(4), 655-665 e618. doi: 10.1016/j.cell.2020.01.008.

Hua, T., Vemuri, K., Pu, M., Qu, L., Han, G.W., Wu, Y., et al. (2016). Crystal Structure of the Human Cannabinoid Receptor CB1. *Cell* 167(3), 750-762 e714. doi: 10.1016/j.cell.2016.10.004.

Israeli, H., Degtjarik, O., Fierro, F., Chunilal, V., Gill, A.K., Roth, N.J., et al. (2021). Structure reveals the activation mechanism of the MC4 receptor to initiate satiation signaling. *Science* 372(6544), 808-814. doi: 10.1126/science.abf7958.

Kim, K., Che, T., Panova, O., DiBerto, J.F., Lyu, J., Krumm, B.E., et al. (2020). Structure of a Hallucinogen-Activated Gq-Coupled 5-HT2A Serotonin Receptor. *Cell* 182(6), 1574-1588 e1519. doi: 10.1016/j.cell.2020.08.024.

Kimura, K.T., Asada, H., Inoue, A., Kadji, F.M.N., Im, D., Mori, C., et al. (2019). Structures of the 5-HT2A receptor in complex with the antipsychotics risperidone and zotepine. *Nat Struct Mol Biol* 26(2), 121-128. doi: 10.1038/s41594-018-0180-z.

Koehl, A., Hu, H., Maeda, S., Zhang, Y., Qu, Q., Paggi, J.M., et al. (2018). Structure of the micro-opioid receptor-Gi protein complex. *Nature* 558(7711), 547-552. doi: 10.1038/s41586-018-0219-7.

Latorraca, N.R., Venkatakrishnan, A.J., and Dror, R.O. (2017). GPCR Dynamics: Structures in Motion. *Chem Rev* 117(1)**,** 139-155. doi: 10.1021/acs.chemrev.6b00177.

Li, X., Hua, T., Vemuri, K., Ho, J.H., Wu, Y., Wu, L., et al. (2019). Crystal Structure of the Human Cannabinoid Receptor CB2. *Cell* 176(3)**,** 459-467 e413. doi: 10.1016/j.cell.2018.12.011.

Lin, X., Li, M., Wang, N., Wu, Y., Luo, Z., Guo, S., et al. (2020). Structural basis of ligand recognition and self-activation of orphan GPR52. *Nature* 579(7797)**,** 152-157. doi: 10.1038/s41586-020-2019-0.

Liu, K., Wu, L., Yuan, S., Wu, M., Xu, Y., Sun, Q., et al. (2020). Structural basis of CXC chemokine receptor 2 activation and signalling. *Nature* 585(7823)**,** 135-140. doi: 10.1038/s41586-020-2492-5.

Liu, W., Chun, E., Thompson, A.A., Chubukov, P., Xu, F., Katritch, V., et al. (2012). Structural basis for allosteric regulation of GPCRs by sodium ions. *Science* 337(6091)**,** 232-236. doi: 10.1126/science.1219218.

Maeda, S., Qu, Q., Robertson, M.J., Skiniotis, G., and Kobilka, B.K. (2019). Structures of the M1 and M2 muscarinic acetylcholine receptor/G-protein complexes. *Science* 364(6440)**,** 552-557. doi: 10.1126/science.aaw5188.

Manglik, A., Kruse, A.C., Kobilka, T.S., Thian, F.S., Mathiesen, J.M., Sunahara, R.K., et al. (2012). Crystal structure of the micro-opioid receptor bound to a morphinan antagonist. *Nature* 485(7398)**,** 321-326. doi: 10.1038/nature10954.

Nojima, S., Fujita, Y., Kimura, K.T., Nomura, N., Suno, R., Morimoto, K., et al. (2021). Cryo-EM Structure of the Prostaglandin E Receptor EP4 Coupled to G Protein. *Structure* 29(3)**,** 252-260 e256. doi: 10.1016/j.str.2020.11.007.

Okada, T., Sugihara, M., Bondar, A.N., Elstner, M., Entel, P., and Buss, V. (2004). The retinal conformation and its environment in rhodopsin in light of a new 2.2 A crystal structure. *J Mol Biol* 342(2)**,** 571-583. doi: 10.1016/j.jmb.2004.07.044.

Rasmussen, S.G., DeVree, B.T., Zou, Y., Kruse, A.C., Chung, K.Y., Kobilka, T.S., et al. (2011). Crystal structure of the beta2 adrenergic receptor-Gs protein complex. *Nature* 477(7366)**,** 549-555. doi: 10.1038/nature10361.

Shimamura, T., Shiroishi, M., Weyand, S., Tsujimoto, H., Winter, G., Katritch, V., et al. (2011). Structure of the human histamine H1 receptor complex with doxepin. *Nature* 475(7354)**,** 65-70. doi: 10.1038/nature10236.

Su, M., Zhu, L., Zhang, Y., Paknejad, N., Dey, R., Huang, J., et al. (2020). Structural Basis of the Activation of Heterotrimeric Gs-Protein by Isoproterenol-Bound beta1-Adrenergic Receptor. *Mol Cell* 80(1)**,** 59-71 e54. doi: 10.1016/j.molcel.2020.08.001.

Suno, R., Lee, S., Maeda, S., Yasuda, S., Yamashita, K., Hirata, K., et al. (2018). Structural insights into the subtype-selective antagonist binding to the M2 muscarinic receptor. *Nat Chem Biol* 14(12)**,** 1150-1158. doi: 10.1038/s41589-018-0152-y.

Thal, D.M., Sun, B., Feng, D., Nawaratne, V., Leach, K., Felder, C.C., et al. (2016). Crystal structures of the M1 and M4 muscarinic acetylcholine receptors. *Nature* 531(7594)**,** 335-340. doi: 10.1038/nature17188.

Toyoda, Y., Morimoto, K., Suno, R., Horita, S., Yamashita, K., Hirata, K., et al. (2019). Ligand binding to human prostaglandin E receptor EP4 at the lipid-bilayer interface. *Nat Chem Biol* 15(1)**,** 18-26. doi: 10.1038/s41589-018-0131-3.

Wang, S., Che, T., Levit, A., Shoichet, B.K., Wacker, D., and Roth, B.L. (2018). Structure of the D2 dopamine receptor bound to the atypical antipsychotic drug risperidone. *Nature* 555(7695)**,** 269-273. doi: 10.1038/nature25758.

Warne, T., Serrano-Vega, M.J., Baker, J.G., Moukhametzianov, R., Edwards, P.C., Henderson, R., et al. (2008). Structure of a beta1-adrenergic G-protein-coupled receptor. *Nature* 454(7203)**,** 486-491. doi: 10.1038/nature07101.

Xia, R., Wang, N., Xu, Z., Lu, Y., Song, J., Zhang, A., et al. (2021). Cryo-EM structure of the human histamine H1 receptor/Gq complex. *Nat Commun* 12(1)**,** 2086. doi: 10.1038/s41467-021-22427-2.

Xu, P., Huang, S., Mao, C., Krumm, B.E., Zhou, X.E., Tan, Y., et al. (2021). Structures of the human dopamine D3 receptor-Gi complexes. *Mol Cell* 81(6)**,** 1147-1159 e1144. doi: 10.1016/j.molcel.2021.01.003.

Yin, W., Zhou, X.E., Yang, D., de Waal, P.W., Wang, M., Dai, A., et al. (2018). Crystal structure of the human 5-HT1B serotonin receptor bound to an inverse agonist. *Cell Discov* 4**,** 12. doi: 10.1038/s41421-018-0009-2.

Yu, J., Gimenez, L.E., Hernandez, C.C., Wu, Y., Wein, A.H., Han, G.W., et al. (2020). Determination of the melanocortin-4 receptor structure identifies Ca(2+) as a cofactor for ligand binding. *Science* 368(6489)**,** 428-433. doi: 10.1126/science.aaz8995.

Zhang, M., Gui, M., Wang, Z.F., Gorgulla, C., Yu, J.J., Wu, H., et al. (2021). Cryo-EM structure of an activated GPCR-G protein complex in lipid nanodiscs. *Nat Struct Mol Biol* 28(3)**,** 258-267. doi: 10.1038/s41594-020-00554-6.

Zhuang, Y., Xu, P., Mao, C., Wang, L., Krumm, B., Zhou, X.E., et al. (2021). Structural insights into the human D1 and D2 dopamine receptor signaling complexes. *Cell* 184(4)**,** 931-942 e918. doi: 10.1016/j.cell.2021.01.027.

Supplementary Figures

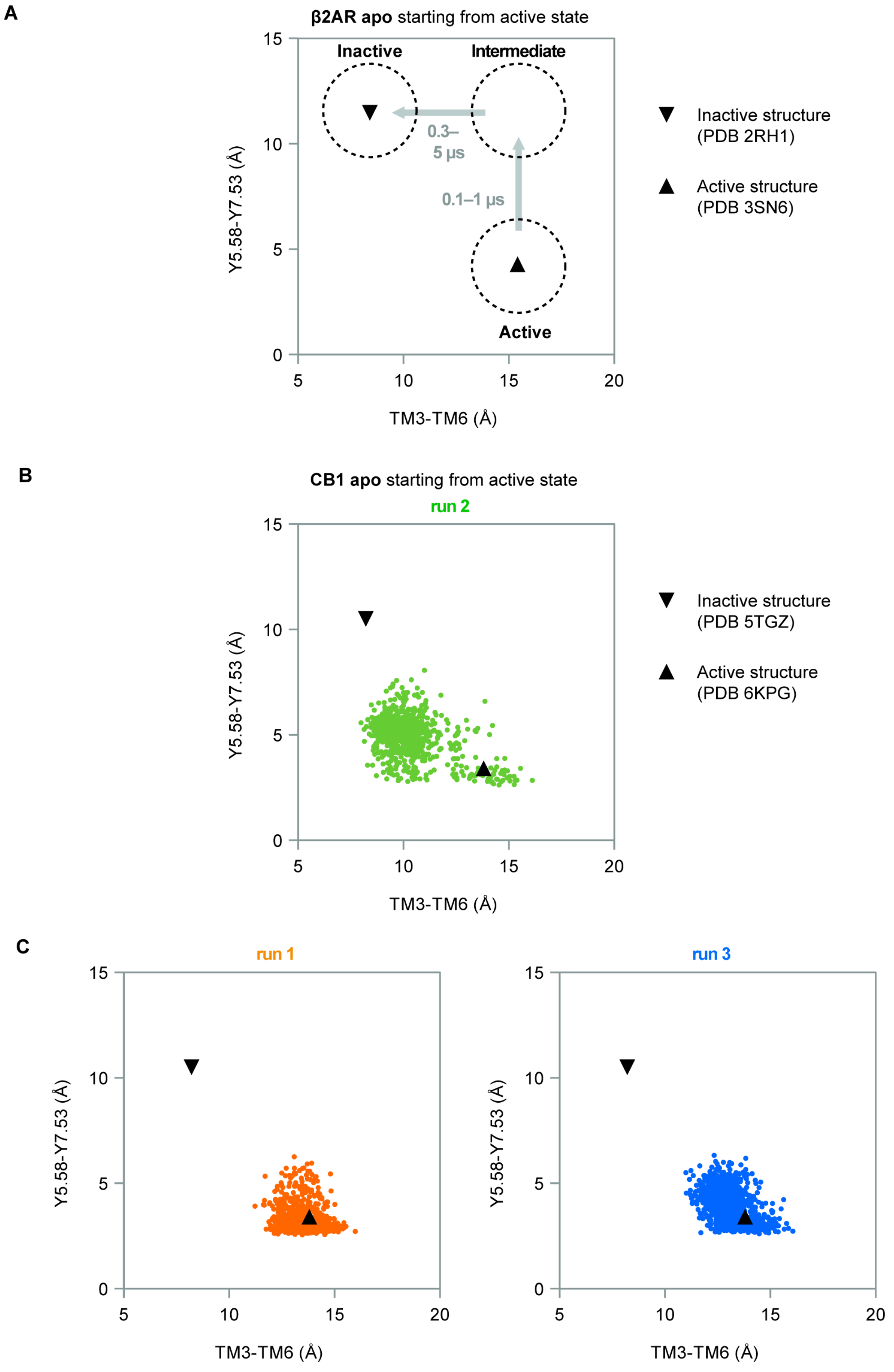

Figure S1. Distribution of TM3-TM6 and Y5.58-Y7.53 in MD simulations. A. Schematic diagram of β2AR deactivation process, showing time scales of transitions (based on results from (Dror et al., 2011)). **B-C.** Results of our simulations of apo CB1 starting from active state: **B.** run 2, with local movement of the cytoplasmic part; **C.** runs 1 and 3, remaining active.

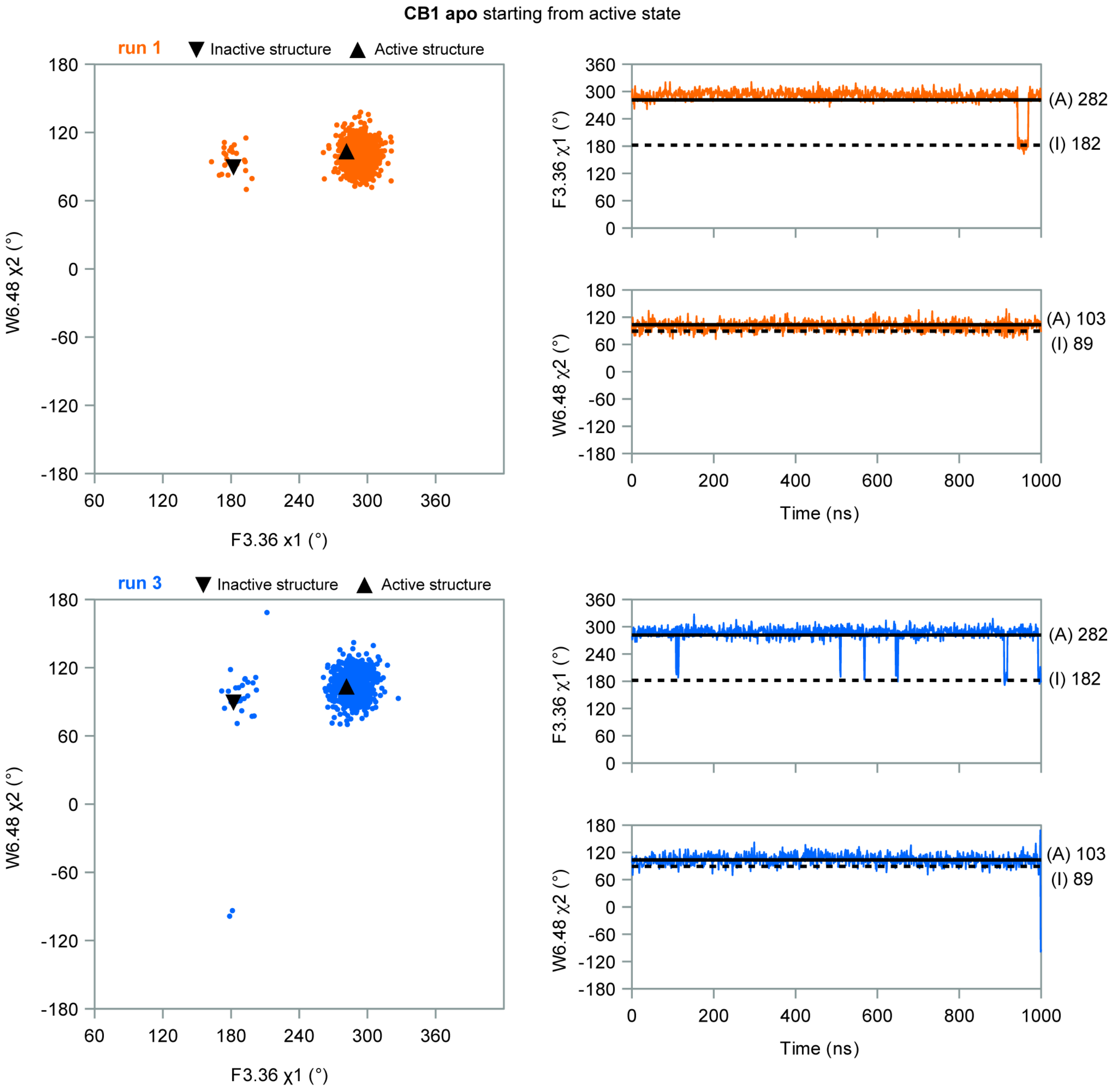

Figure S2. F200$^{3.36}$/W356$^{6.48}$ side-chain conformations during MD simulations of apo CB1 starting from active state in runs 1 and 3 (additional information for Figure 2C). Left panel: Distribution of dihedral angles. Right panel: F200$^{3.36}$ χ1 and W356$^{6.48}$ χ2 during simulation.

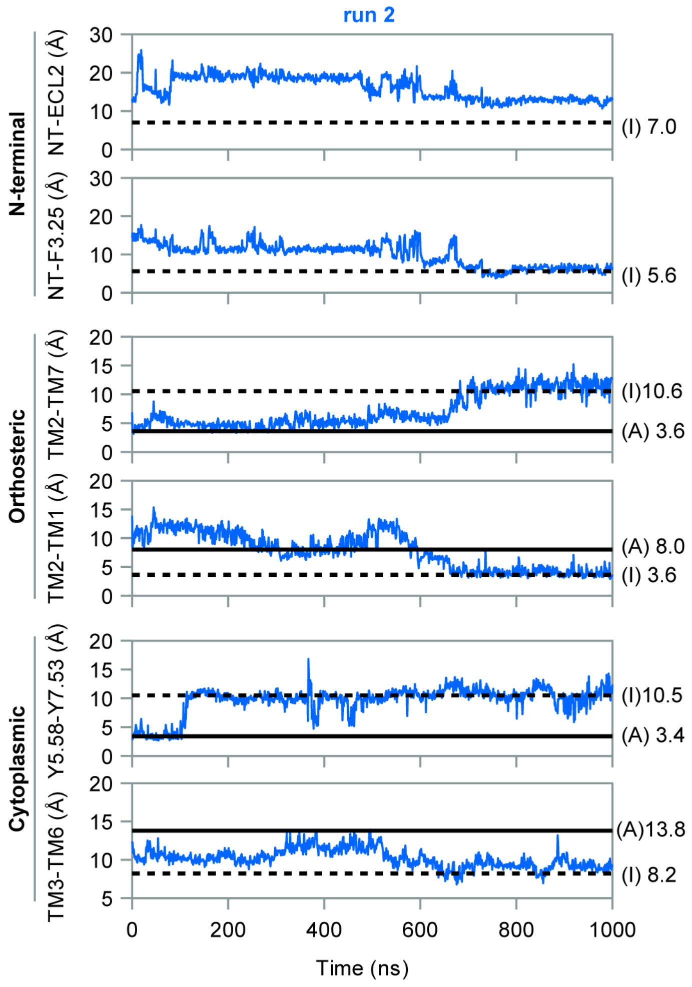

Figure S3. MD simulation results of CB1 double-mutant F200A/W356A starting from active state (additional information for Figure 3B). Indicators for movements at different parts of CB1 in run 2, showing deactivation transitions started at the cytoplasmic end and finalized at the extracellular end. The two N-terminal indicators could not be measured in the active structure because $F102^{N\text{-term}}$ was not solved.

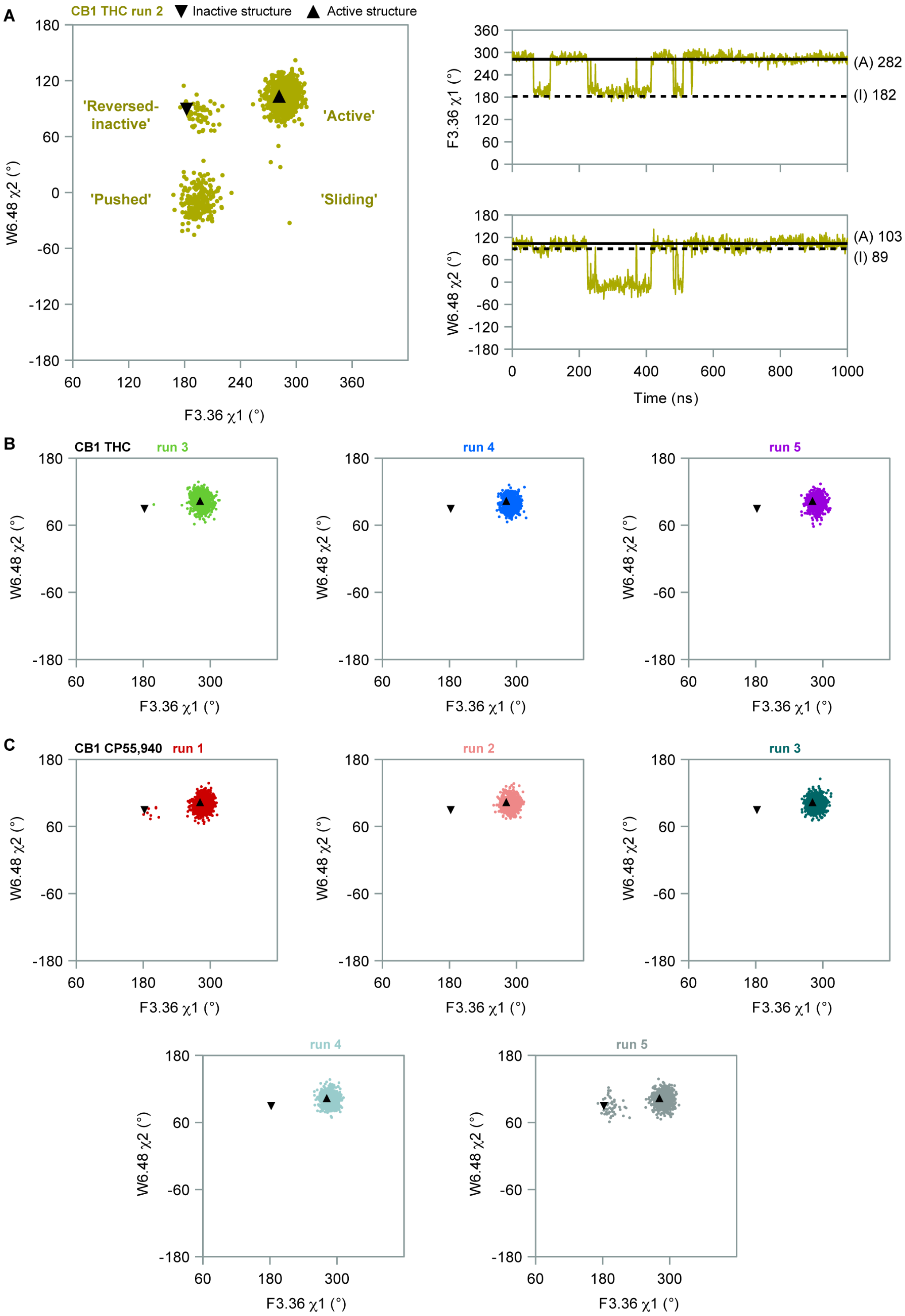

Figure S4. F200$^{3.36}$/W356$^{6.48}$ side-chain conformations during MD simulations of CB1 bound with THC and CP55,940 (additional information for Figure 4). A. Movements of F200$^{3.36}$ and W356$^{6.48}$ in THC-bound CB1 run 2. Left panel: Distribution of dihedral angles showing configurations of F200$^{3.36}$/W356$^{6.48}$. Right panel: F200$^{3.36}$ $\chi 1$ and W356$^{6.48}$ $\chi 2$ during simulation. **B.** Distribution of dihedral angles of F200$^{3.36}$/W356$^{6.48}$ in THC-bound CB1 runs 3–5. **C.** Distribution of dihedral angles of F200$^{3.36}$/W356$^{6.48}$ in CP55,940-bound CB1 runs 1–5.

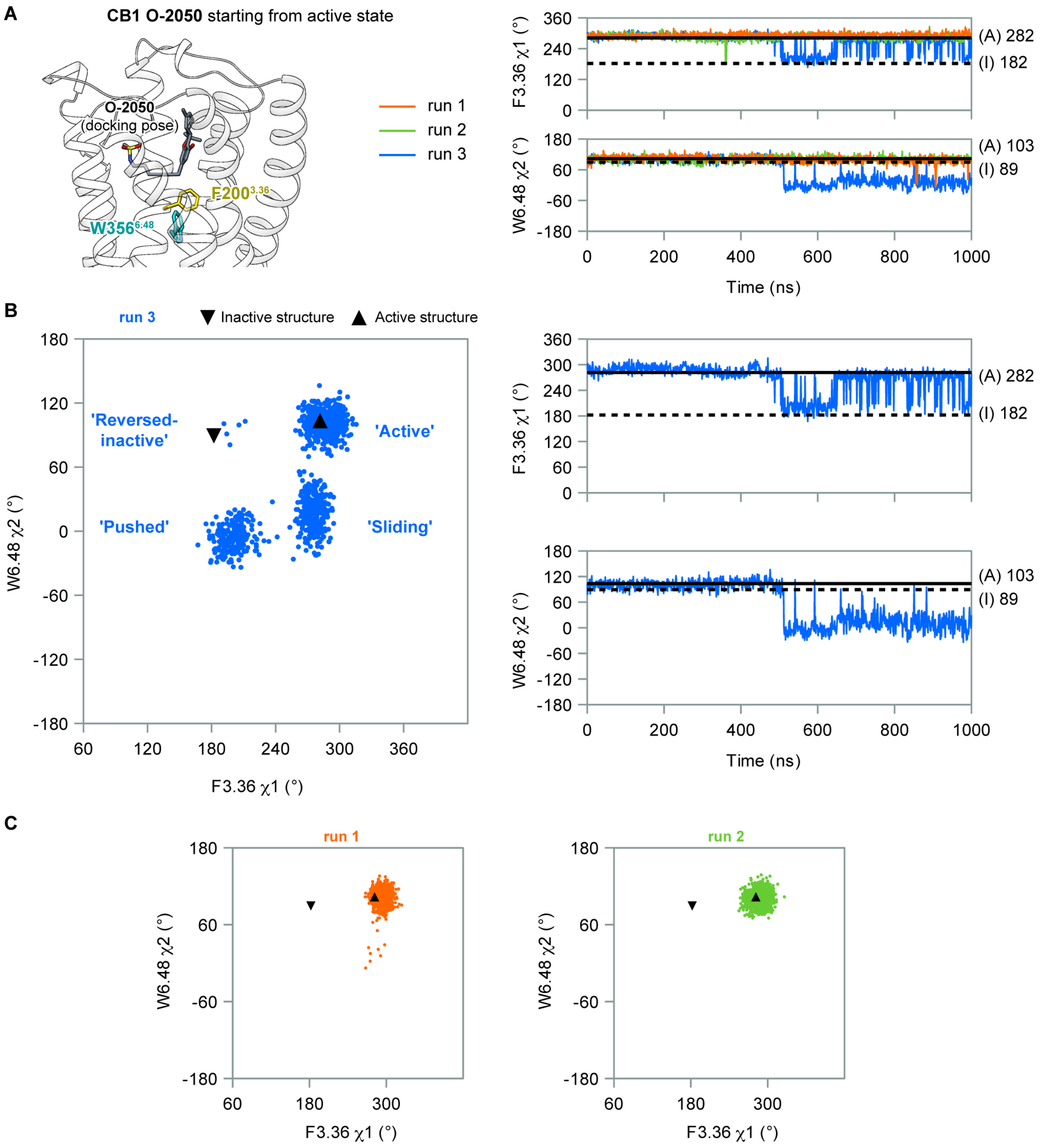

Figure S5. F200$^{3.36}$/W356$^{6.48}$ side-chain conformations during MD simulations of CB1 bound with neutral antagonist O-2050. A. Fluctuations of F200$^{3.36}$ and W356$^{6.48}$ side-chains were captured in run 3. **B.** Movements of F200$^{3.36}$ and W356$^{6.48}$ in run 3. Left panel: Distribution of dihedral angles showing configurations of F200$^{3.36}$/W356$^{6.48}$. Right panel: F200$^{3.36}$ χ1 and W356$^{6.48}$ χ2 during simulation. **C.** Distribution of dihedral angles of F200$^{3.36}$/W356$^{6.48}$ in O-2050-bound CB1 runs 1 and 2.

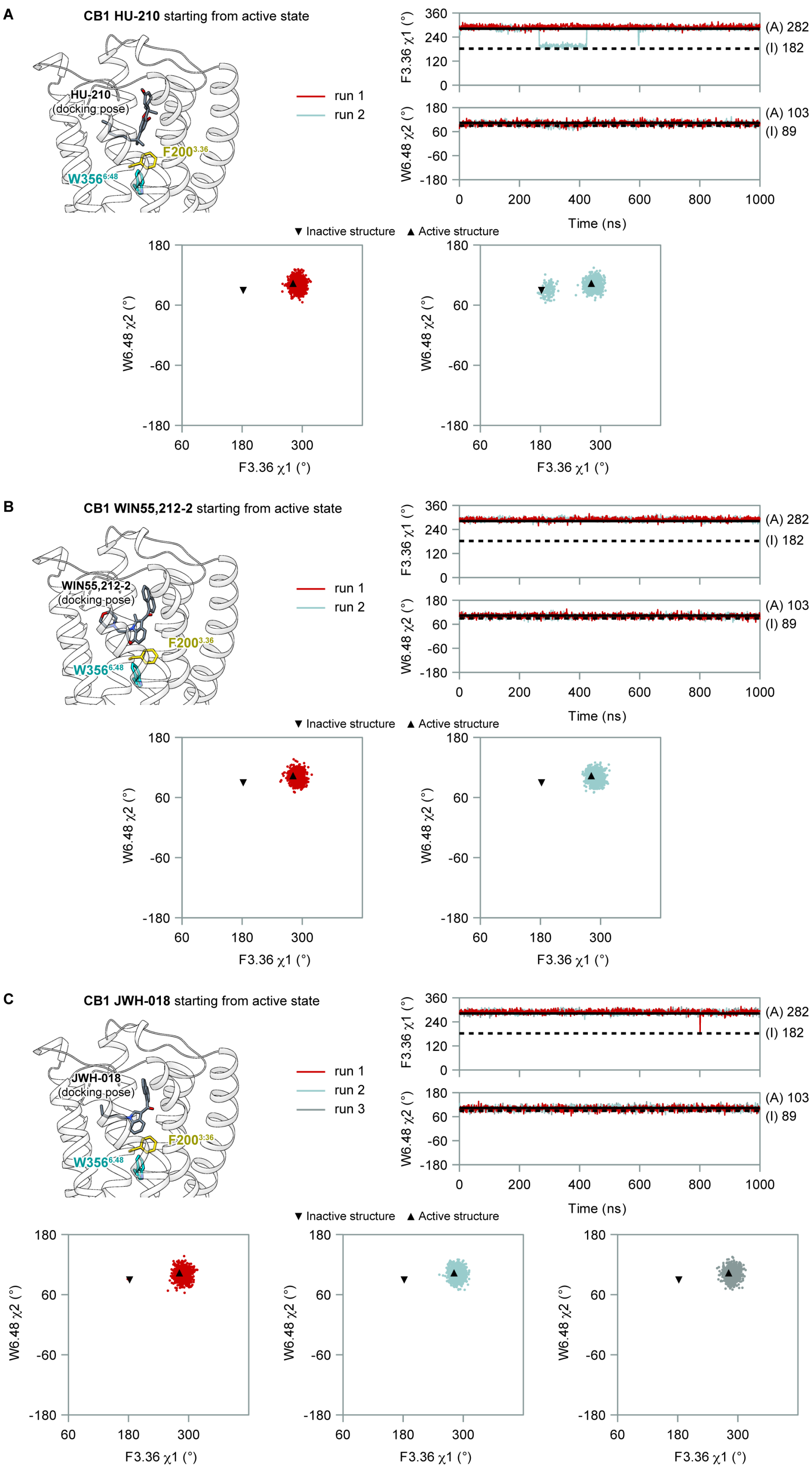

Figure S6. F200$^{3.36}$/W356$^{6.48}$ side-chain conformations during MD simulations of CB1 bound with full agonists, HU-210 (A), WIN55,212-2 (B), and JHW-018 (C).

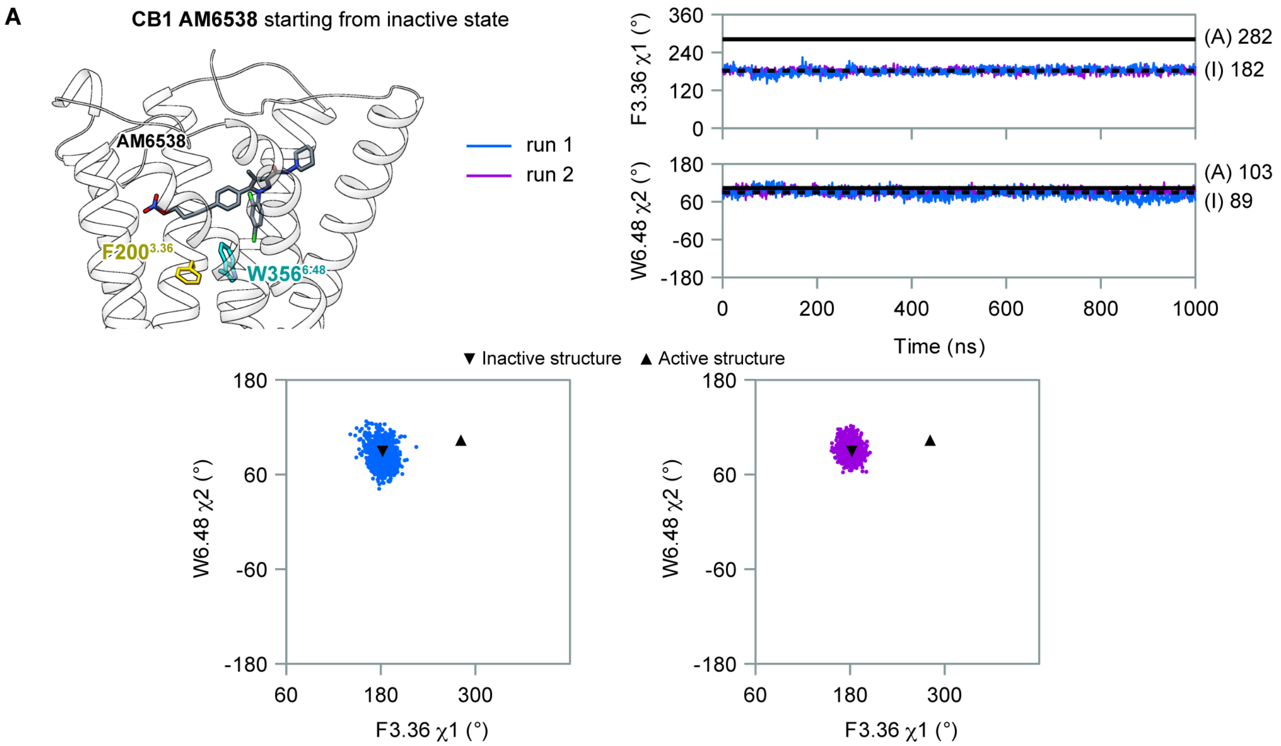

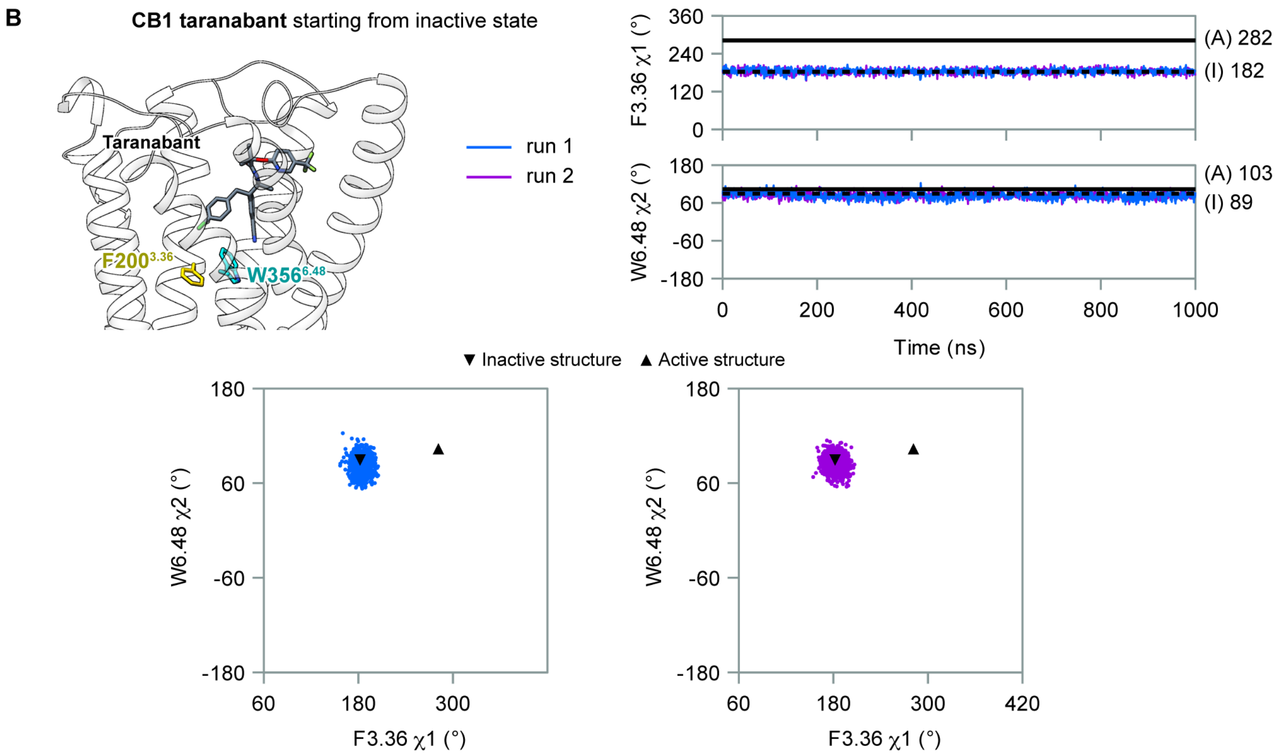

Figure S7. F200$^{3.36}$/W356$^{6.48}$ side-chain conformations during MD simulations of CB1 bound with inverse agonists, AM6538 (A) and taranabant (B).

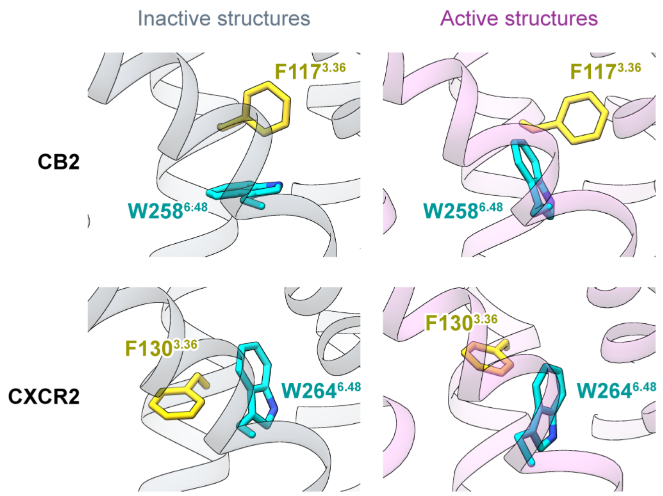

Figure S8. CB2 and CXCR2 do not have 'twin toggle switch' of F3.36/W6.48.

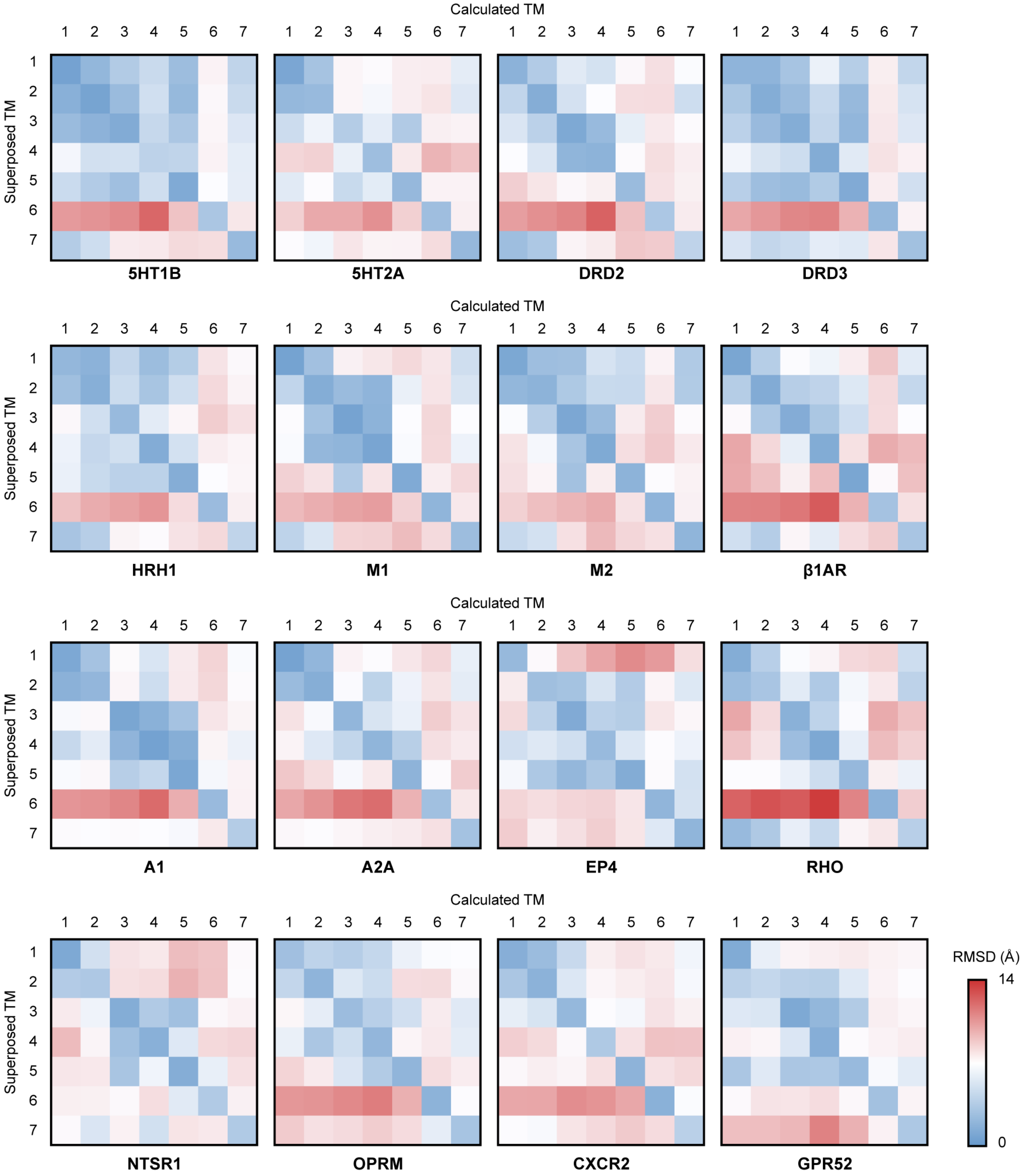

Figure S9. 7 × 7 RMSD matrices of inactive/active structure pairs for 16 class A GPCRs (additional information for Figure 5C).
